# Pituitary function at presentation and following therapy in patients with non-functional pituitary macroadenomas: a single centre retrospective cohort study

**DOI:** 10.1007/s12020-023-03434-3

**Published:** 2023-06-30

**Authors:** Ziad Hussein, Hani J. Marcus, Joan Grieve, Neil Dorward, Michael Kosmin, Naomi Fersht, Pierre Marc Bouloux, Zane Jaunmuktane, Stephanie E. Baldeweg

**Affiliations:** 1 Department of Diabetes and Endocrinology, Sheffield Teaching Hospitals, Sheffield, UK; 2 Division of Medicine, University College London, London, UK; 3 Department of Endocrinology, University College London Hospitals, London, UK; 4 Department of Neurosurgery, National Hospital for Neurology and Neurosurgery, London, UK; 5 Department of Clinical Oncology, University College London Hospitals, London, UK; 6 Centre for Neuroendocrinology, Royal Free Campus, University College Medical School, University College London, London, UK; 7 Institute of Neurology, University College London, London, UK

**Keywords:** Non-functioning Pituitary Adenoma, Hypopituitarism, Transsphenoidal Surgery, Radiotherapy

## Abstract

**Background:**

Non-functioning pituitary macroadenomas (NFPMs) may present with hypopituitarism. Pituitary surgery and radiotherapy pose an additional risk to pituitary function.

**Objectives:**

To assess the incidence of hypopituitarism at presentation, the impact of treatment, and the likelihood of endocrine recovery during follow-up.

**Methods:**

All patients treated surgically with and without radiotherapy for NFPMs between 1987 and 2018 who had longer than six months follow-up were identified. Demographics, presentation, investigation, treatment, and outcomes were collected.

**Results:**

In total, 383 patients were identified. The median age was 57 years, with a median follow-up of 8 years. Preoperatively, 227 patients (227/375; 61%) had evidence of at least one pituitary deficiency. Anterior panhypopituitarism was more common in men (*p* = 0.001) and older patients (*p* = 0.005). Multiple hormone deficiencies were associated with large tumours (*p* = 0.03). Patients treated with surgery and radiotherapy had a higher incidence of all individual pituitary hormone deficiency, anterior panhypopituitarism, and significantly lower GH, ACTH, and TSH deficiencies free survival probability than those treated with surgery alone. Recovery of central hypogonadism, hypothyroidism, and anterior panhypopituitarism was also less likely to be reported in those treated with surgery and radiotherapy. Those with preoperative hypopituitarism had a higher risk of pituitary impairment at latest review than those presented with normal pituitary function (*p* = 0.001).

**Conclusion:**

NFPMs are associated with a significant degree of hypopituitarism at time of diagnosis and post-therapy. The combination of surgery and radiotherapy is associated with a higher risk of pituitary dysfunction. Recovery of pituitary hormone deficit may occur after treatment. Patients should have regular ongoing endocrine evaluation post-treatment to assess changes in pituitary function and the need for long-term replacement therapy.

## Introduction

Non-functioning pituitary adenomas are benign tumours of the adenohypophysis with gonadotropin-expressing subtype accounting for up to 80% of these tumours [1–3]. These neoplasms vary in clinical presentation from an incidental finding on neuroimaging to large macroadenomas damaging the pituitary gland, optic apparatus, and surrounding neurological structures [4, 5]. Hypopituitarism can be the presenting manifestation in many patients; however, the process of developing pituitary hormone deficiency is often latent and asymptomatic.

The risk of developing partial and complete hypopituitarism in patients with NFPMs is increased after surgical resection and radiotherapy. Moreover, in selected patients, there is a recovery of pituitary function [6]. Long term and regular screening for hypothalamic-pituitary insufficiency after NFPMs treatment is therefore of crucial importance to provide appropriate replacement therapy and to prevent short and long term patient morbidity and mortality [7–9].

In this study, we investigated the course of pituitary dysfunction in NFPMs following surgical resection with and without radiotherapy. The aims were to assess the incidence of hypopituitarism pre-operatively in patients with NFPMs and following surgery with and without radiotherapy and the likelihood of recovery of pituitary function post-treatment during follow-up.

## Methods

Ethics approval to conduct this study was obtained from Westminster Research Ethics Committee on 07/04/2020. The Strengthening the Reporting of Observational Studies in Epidemiology (STROBE) Statement was used in the preparation of this section of the manuscript [10]. This study was conducted as a single-centre cohort study. It included all patients who underwent surgical resection for non-functioning pituitary macroadenomas between 1987 and 2018, with a follow-up duration of more than 6 months. The study was conducted at the National Hospital for Neurology and Neurosurgery in London, one of the busiest neurosurgical centres and the largest pituitary centre in the United Kingdom. Surgical resection was performed mainly by three experienced neurosurgeons (MP, JG and ND). A retrospective review of electronic case notes was performed.

### Data collection

Data on patients’ demographics, clinical presentation, treatment modalities and details of endocrine testing were collected.

The diagnosis of NFPMs was based on the absence of clinical and biochemical evidence of pituitary hormone hypersecretion and/or expression of pituitary adenoma markers on immunohistological analysis of surgically resected pituitary specimens.

### Endocrine function assessment

Patients were diagnosed with severe growth hormone (GH) deficiency on provocative testing, insulin tolerance test (ITT) or glucagon stimulation test (GST), with peak GH response <3 µg/L. In addition, patients with three or more anterior pituitary hormone deficiencies and low IGF1 in the absence of liver disease or malnourishment were also considered GH deficient [11, 12]. Recovery of growth hormone axis following therapy was reported in those who achieved peak GH level more than the diagnostic cut-off of dynamic testing.

Our centre’s diagnostic criteria for secondary adrenal insufficiency for this study were: 0800–0900 a.m. cortisol level of less than 100 nmol/L and suboptimal peak cortisol response to dynamic testing. Patients with peak cortisol of less than 550 nmol/L and 415 nmol/L, in those who underwent ITT and SST, before and after September 2015, respectively, were considered to have ACTH deficiency [13–16]. Peak cortisol less than 450 nmol/L on GST was also diagnostic of ACTH impairment [17]. Furthermore, patients with 0800–0900 a.m. cortisol of less than 350 nmo/L during the immediate postoperative phase were commenced on glucocorticoid therapy until full endocrine assessment 6–8 weeks post-surgery. Recovery of the hypothalamic-pituitary-adrenal axis was recorded in those patients with basal morning cortisol of above 400 nmol/L and in those who achieved peak cortisol higher than the diagnostic threshold of provocative testing.

Dynamic testing for GH and ACTH deficiency was not performed when there was clear evidence of both axes impairment on baseline biochemical assessment.

Patients with a deficiency of thyrotropin-stimulating hormone (TSH) were reported deficient when a free T4 dropped below the normal reference range with inappropriately normal or low TSH. Patients with primary hypothyroidism were excluded from being TSH deficient. Recovery of this axis was recorded in patients who maintained normal TSH and free T4 without levothyroxine replacement therapy.

Central hypogonadism was defined as follows: men were deficient if they were receiving testosterone therapy or if their early morning testosterone level was less than 8 nmol/L in the presence of inappropriately normal or low serum gonadotropins. In premenopausal women, amenorrhoea or oligomenorrhoea with low oestradiol less than 90 nmol/L and inappropriately normal or low serum gonadotrophins were diagnosed with hypogonadotropic hypogonadism. In addition, women who received oestrogen replacement in the form of hormone replacement therapy were also classified as deficient in gonadotrophins. Furthermore, postmenopausal women with non-elevated serum gonadotrophins were documented as gonadotrophin deficient. Regaining gonadotroph function following surgery was considered when there was a normal gonadotrophins level with normal testosterone in men and normal oestradiol in women up to the age of 55 years without replacement therapy. In postmenopausal women, raised gonadotrophins were considered as an indication of gonadotroph recovery.

Anterior hypopituitarism was reported when there was a loss of three or more anterior pituitary hormones.

Central diabetes insipidus was diagnosed in those with urine output of more than 3 l or 50 ml/kg/24 h, urine osmolality less than 800 mOsm/kg, and an increase in urine osmolality by >50% from baseline following desmopressin administration on water deprivation test. In the immediate postoperative period, polyuria of more than 300 mls for three or more consecutive hours and low urine specific gravity with hypernatremia and/or raised serum osmolality was diagnostic for central diabetes insipidus. Patients referred to our centre on replacement therapy commenced at their local hospitals were considered deficient in the relevant pituitary hormone.

### Statistical analysis

Basic data were evaluated using descriptive statistics. Mean and standard deviation were used to describe continuous variables. Median and interquartile range (IQR) were used to describe data not normally distributed. Exact Fisher’s test was used to compare categorical variables, including the trend in postoperative sodium level. Hypopituitarism-free survival probability curves were generated by Kaplan–Meier method and the evaluations of the differences in the various sub-groups were done by the log-rank test. The Spearman analysis test was used to measure the association between continuous and nominal variables. IBM SPSS statistics version 28 was used in this study.

## Results

### Patients’ characteristics, clinical, biochemical and radiological features at presentation

Patients’ demographics and non-functioning pituitary macroadenoma tumour radiological characteristics are shown in Table 1. In total, 383 patients were identified for this study; 256 (256/383; 67% male) with a median follow-up duration of 8 years (IQR 5–10 years). The median age for the cohort was 57 years (IQR 47–66 years).

**Table 1:** Patients’ demographics and non-functioning pituitary macroadenoma tumour radiological characteristics

Median age (IQR)	57 years (47–66)
Male	256/383; 67%
Female	127/383; 33%
Median tumour volume (IQR)	6.8 cm^3^ (4.0–12.7)
Macroadenoma with or without suprasellar extension but not touching the optic chiasm	14/383; 4%
NFPM abutting or compressing the optic apparatus	357/383; 93%
Cavernous Sinus invasion	113/383; 30%
Sphenoid invasion	21/383; 5%
Clivus invasion	9/383; 2%
Extension to the lateral ventricle	5/383; 1%
Invasion of the nasopharynx	2/383; <1%
Temporal lobe invasion	1/383; <1%

*IQR* interquartile range

The leading presenting symptom was visual dysfunction (228/383; 60%). Initial clinical presentation due to hypopituitarism occurred in 58 patients (58/377, 15%). On endocrine evaluation, 227 patients (227/375; 61%) had evidence of deficiency of at least one pituitary hormone; 70 patients (70/227; 31%) had single pituitary hormone deficiency and 157 (157/227; 69%) patients presented with multiple hormones deficiencies. One-third of the patients (115/375; 31%) had GH deficiency, and hypogonadotropic hypogonadism was recorded in 161 patients (161/375; 43%). Dysfunction of the hypothalamic-pituitary-adrenal axis was documented in 132 patients (132/375; 36%), while 157 patients (157/375; 42%) had secondary hypothyroidism. Anterior panhypopituitarism was reported in 100 patients (100/375; 27%).

Anterior panhypopituitarism at presentation was more common in men (83/251; 33%) than women (17/124; 14%) (*p* = 0.001) and observed more with increasing age with a median age of 62 years versus 56 years for those with no preoperative anterior panhypopituitarism (*p* = 0.005). On preoperative imaging, those with multiple pituitary hormone deficiencies had significantly larger NFPMs (median volume = 6.6 cm^3^ [IQR 4.6–13 cm^3^] than those with one hormone deficiency and those with normal pituitary function (median volume = 6.3 cm^3^ [IQR 3.1–10.5])(*p* = 0.03). Cavernous sinus invasion did not influence the development of hypopituitarism, (73/113; 65%) with cavernous sinus invasion versus without (151/258; 59%) (*p* = 0.3). Sixty-six patients had pituitary macroadenoma detected incidentally on radiological imaging (66/383; 17%) and secondary to headache in 41 patients (41/383; 11%). Twelve patients (12/377; 3%) were admitted due to pituitary apoplexy.

### Treatment

With regards to treatment modality, 318 patients (318/383; 83%) were treated with surgery alone, and 65 patients (65/383; 17%) received radiotherapy at some point after surgery. External beam irradiation of 50.4 Gray in 28 daily fractions was delivered to 63 patients, while two patients were treated with Gamma Knife radiosurgery. Patients treated with surgery and radiotherapy were younger (median age = 53 years) than those treated with surgery alone (median age = 59 years) (*p* = 0.004). With regards to histological data, 271 patients (371/383; 97%) had gonadotroph adenomas, and 12 patients (12/383; 3%) had plurihormonal adenomas; all were clinically and biochemically non-functioning.

### Pituitary function after receiving therapy for NFPMs

#### New onset of hormone deficiency in patients with normal endocrine function at presentation

Dynamic testing was performed to assess GH and ACTH deficiency in 247 patients (247/383; 64%). The incidence of new individual pituitary hormone deficiency and anterior panhypopituitarism was significantly higher among patients who received surgery and radiotherapy than those treated with surgery alone (Table 2). The risk of developing endocrine insufficiency in those who received irradiation varied from 3 times in the case of FSH/LH dysfunction to 9 times in central hypothyroidism. The timing of new-onset pituitary hormone deficiencies is demonstrated in Table 3.

**Table 2:** New onset of pituitary hormone deficit following surgery and radiotherapy in patients with non-functioning pituitary macroadenomas

	Total	Surgery	Surgery and Radiotherapy	*P* value	Odds ratio
GH	74/260 (28%)	46/217 (21%)	28/43 (65%)	<0.001	6.9
FSH/LH	48/214 (23%)	33/179 (19%)	15/35 (43%)	0.004	3.3
ACTH	57/243 (23%)	34/203 (17%)	23/40 (58%)	<0.001	7.7
TSH	64/218 (29%)	41/186 (22%)	23/32 (72%)	<0.001	9
Anterior panhypopituitarism	61/275 (22%)	34/229 (15%)	27/46 (59%)	<0.001	8.1

*GH* growth hormone, *FSH* follicle stimulating hormone, *LH* luteinising hormone, *ACTH* adrenocorticotropic hormone, *TSH* thyrotroph stimulating hormone

**Table 3:** Timeline of developing pituitary hormone deficiency and recovery following primary surgery for the full cohort with non-functioning pituitary macroadenomas. The median time is expressed in months

Development of pituitary hormone deficiency	Median time (IQR)
GH	15 months (2–53)
ACTH	3 months (1–29)
FSH/LH	39 months (10–92)
TSH	14 months (3–52)
Pituitary hormone recovery	
GH	11 months (3–32)
ACTH	8 months (4–17)
FSH/LH	10 months (6–26)
TSH	9 months (4–33)

*GH* growth hormone, *FSH* follicle stimulating hormone, *LH* luteinising hormone, *ACTH* adrenocorticotropic hormone, *TSH* thyrotroph stimulating hormone, *IQR* interquartile range

#### Pituitary hormone recovery after treatment in patients presented with hypopituitarism

When comparing pituitary function at latest review with baseline levels at presentation for those presented with pituitary dysfunction, the pituitary-adrenal axis recovered in 41 patients (41/132; 31%), whereas reversal of growth hormone deficiency occurred in 28 patients (28/115; 24%) (Table 4). Normal gonadal function was observed in 36 patients (36/160; 23%), and secondary hypothyroidism resolved in 20 patients (20/157; 13%). Improvement in anterior panhypopituitarism was reported in 32 patients (32/100; 32%). Across the full cohort, younger age was observed to have a higher rate of improvement in gonadotropin deficiency (*p* = 0.004), secondary hypocortisolism (*p* = 0.01) and anterior panhypopituitarism (*p* = 0.006). Gender and evidence of complete resection of NFPMs on postoperative MRI scans were not related to pituitary recovery.

**Table 4:** Pituitary recovery after surgical resection and irradiation of non functioning pituitary macroadenomas

	Total	Surgery	Surgery and radiotherapy	*P* value
GH	28/115 (24%)	27/99 (27%)	1/16 (6%)	0.1
FSH/LH	36/160 (23%)	35/137 (26%)	1/23 (4%)	0.02
ACTH	41/132 (31%)	38/113 (33%)	3/19 (21%)	0.2
TSH	18/157 (12%)	18/130 (14%)	0/27 (0%)	0.03
Anterior panhypopituitarism	32/100 (32%)	31/87 (36%)	1/13 (8%)	0.06

*GH* growth hormone, *FSH* follicle stimulating hormone, *LH* luteinising hormone, *ACTH* adrenocorticotropic hormone, *TSH* thyrotroph stimulating hormone

The likelihood of improvement in gonadotropins, TSH deficiencies, and anterior panhypopituitarism for patients treated with surgery and radiotherapy was significantly less than for those who underwent surgery only. Notably, none of TSH deficient patients regained normal thyroid function post-irradiation. The timing of pituitary hormone recovery is shown in Table 3.

#### Hypopituitarism for the full cohort at the latest follow-up

At latest endocrine evaluation, 105 patients (105/383; 27%) had no evidence of pituitary dysfunction, while 278 (278/383; 73%) patients had evidence of pituitary deficiency; 80 patients (80/278; 29%) had single hormone deficit and 198 patients (198/278; 71%) had two or more hormone impairment. Patients presented with any degree of hypopituitarism (*n* = 227) at the time of diagnosis of NFPMs had a higher risk of having pituitary impairment at the latest follow-up when compared to those presented with normal pituitary function (*n* = 148), (202/227; 89%) versus (70/148; 47%) (*p* = 0.001), respectively.

**Fig. 1:**
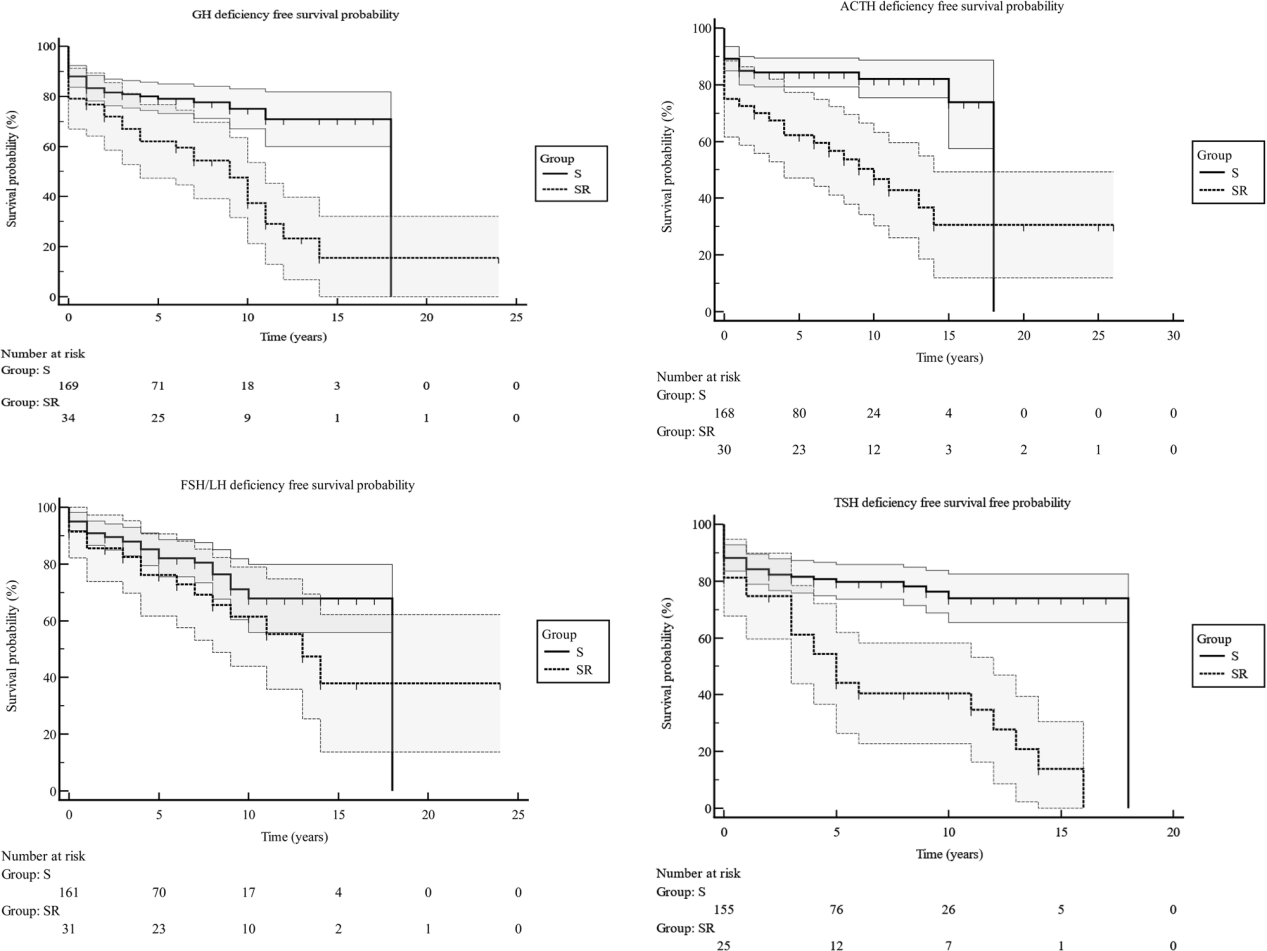
The probability of pituitary deficiency free survival following surgery and radiotherapy for patients with non-functioning pituitary macroadenomas using Kaplan-Meier analysis. GH Growth Hormone, FSH Follicle Stimulating Hormone, LH Luteinising Hormone, ACTH Adrenocorticotropic Hormone, TSH Thyrotroph Stimulating Hormone, S Surgery group, SR Surgery and Radiotherapy group

In total, 165 patients (165/383; 43%) were GH deficient (Table 5). Secondary hypogonadism was reported in 178 patients (178/383; 46%), while 156 patients (156/383; 41%) suffered secondary hypocortisolism. Thyroid dysfunction was recorded in 206 patients (206/383; 54%), and 133 patients (133/383; 34%) had anterior panhypopituitarism. Twenty-three patients (23/383; 6%) developed permanent cranial diabetes insipidus. Anterior panhypopituitarism was more commonly observed in men (102/256; 40%) than women (31/127; 24%) (*p* = 0.003) and in those presented with anterior panhypopituitarism (68/100; 84%) at time of diagnosis than those presented without (61/275; 22%) (*p* = 0.001). Patients who received postoperative pituitary radiotherapy had a greater degree of partial and complete hypopituitarism than those treated with surgery alone. In addition, those who underwent surgery alone had higher GH, ACTH and TSH deficiency free survival probability than those who received surgery and radiotherapy (Fig. 1).

**Table 5:** Pituitary hormones dysfunction for the full cohort with non-functioning pituitary macroadenomas at latest clinic review

	Total	Surgery	Surgery and radiotherapy	*P* value
GH	165/383 (43%)	118/318 (37%)	47/65 (72%)	<0.0001
FSH/LH	178/383 (46%)	136/318 (43%)	42/65 (65%)	0.001
ACTH	156/383 (41%)	111/318 (35%)	45/65 (69%)	<0.0001
TSH	206/383 (54%)	151/318 (47%)	55/65 (85%)	<0.0001
Anterior panhypopituitarism	133/383 (35%)	90/318 (28%)	43/65 (62%)	0.0001

*GH* growth hormone, *FSH* follicle stimulating hormone, *LH* luteinising hormone, *ACTH* adrenocorticotropic hormone, *TSH* thyrotroph stimulating hormone

## Discussion

### Principal findings

In this study, we describe the extent of preoperative endocrine dysfunction of NFPMs, the impact of surgery and radiotherapy on pituitary function, and the likelihood of hormone deficit recovery during follow-up. We report the following principal findings: (1) hypopituitarism commonly occurs in patients with NFPMs without relevant clinical symptoms before presentation; (2) patients treated with surgery and radiotherapy had a higher rate of permanent pituitary dysfunction than those that underwent surgery alone; (3) Recovery of pituitary function was significantly less frequent in patients who received surgery and radiotherapy (4) Younger patients had a higher degree of endocrine recovery after NFPMs treatment.

### Comparison with other studies

Mechanical compression and damage of the adenohypophysis by the enlarging NFPMS can lead to endocrine dysfunction field [18]. In addition, reduction of the pituitary portal circulation due to mass effect has been speculated to cause hypopituitarism [19]. A substantial number of our patients had evidence of pituitary hormone deficiency on laboratory assessment at presentation despite being asymptomatic. Hypopituitarism often develops insidiously and remains undiagnosed until the patient undergoes full clinical and endocrine evaluation field [18]. Interestingly in this study, male gender, older age, and NFPMs extending beyond the sella turcica were associated with a higher rate of anterior panhypopituitarism at presentation. Jahangiri et al. [20] reported a correlation between any degree of preoperative hypopituitarism and old age, gender and large pituitary adenomas but not linked to complete hypopituitarism. Zhang et al. [6] recorded significantly lower preoperative levels of thyroxine, GH, IGF-1, FSH, and LH in patients with giant NFPAs compared to those with macroadenoma, demonstrating the impact of larger NFPAs on pituitary function. Our cohort with reported partial and complete hypopituitarism at diagnosis is in line with other studies in the literature [6, 8, 18]. The agreed sequential pattern of developing pituitary hormone deficiency in patients with pituitary adenomas at presentation is often debated. The classical description of losing GH axis followed by gonadotroph, thyrotroph and adrenocorticotroph impairment is generally agreed on [21–24]; however, many studies demonstrate a different pattern of pituitary hormone insufficiencies [6, 25, 26]. Carosi et al. [25] showed that central hypogonadism was more frequently observed in NFPMs, followed by GH, ACTH and TSH deficiencies. A similar pattern of pituitary impairment was also demonstrated by Ferrante et al. [26].

The definitive diagnosis of GH insufficiency at diagnosis of pituitary adenomas requires provocative testing, which is often performed in selected patients only when there is an intention to treat; this may lead to underdiagnosis of the condition. Yuen et al. [27] demonstrated that 50% of their 38 patients with non-functioning pituitary microadenomas and normal IGF1 levels had GH insufficiency using growth hormone releasing hormone-arginine test. In contrast, adrenal insufficiency is investigated extensively, at presentation and after therapy, to establish the presence of hypocortisolism and to commence glucocorticoid therapy, when appropriate, to prevent adrenal crises. This study has a higher incidence of central adrenal insufficiency than reported in the literature. Our hospital is a large tertiary centre that receives a high volume of referrals from the local district hospitals. Patients who were referred from these hospitals while on glucocorticoid replacement were considered to have adrenal insufficiency. This could be one of the potential causes. The lower frequency of GH deficiency at presentation in this study may not reflect the overall incidence of the condition, as GH deficiency diagnosis requires dynamic tests, as discussed above, these may only be performed for selected cases before surgery.

Perioperative glucocorticoid replacement was traditionally used in all patients undergoing surgery for suprasellar and extrasellar tumours to prevent adrenal crisis, but this approach carries the risk of potential exposure to steroid side effects, including hyperglycaemia, peptic ulcers, sleep disturbance and osteoporosis. In addition, assessing adrenal function following surgery in patients treated empirically with exogenous steroids might be challenging and difficult to interpret. Many studies demonstrated the safety of performing surgical resection without glucocorticoid cover in those with normal hypothalamic–pituitary–adrenal axis before surgery [28, 29]. In our centre, patients with confirmed ACTH deficiency before surgery were commenced on glucocorticoid therapy and received parenteral hydrocortisone 50–100 mg 6 h perioperatively. In addition, those patients received supraphysiological or stress doses of glucocorticoid therapy in the immediate postoperative phase. Two of the three neurosurgeons did not commence perioperative glucocorticoids in patients with normal adrenal function, while one surgeon’s approach was starting steroid cover in all patients perioperatively. 0800 a.m. cortisol level was assessed 48 h post-surgery in all patients after withdrawing steroid treatment for at least 18 h when applicable. Morning cortisol less than 350 nmol/L reflected central hypocortisolism in our centre and was managed with glucocorticoid replacement until definitive dynamic testing was performed 6–8 weeks following surgery.

The occurrence of new pituitary dysfunction post-therapy can vary according to treatment modality. Postoperatively, the likelihood of developing pituitary dysfunction has been linked to many factors: the operating neurosurgeon, pituitary tumour size, the degree of surgical manipulation, and the need for multiple surgeries to deal with recurrent disease [30]. Similarly, the occurrence of new-onset pituitary insufficiency post-radiotherapy depends on many factors; radiation dose, technique and follow-up duration [31, 32]. It has been estimated that around 30–60% of the patients may develop endocrine dysfunction after pituitary external beam irradiation [32]. We demonstrated that the combination of surgery and radiotherapy is associated with a significantly higher risk of pituitary dysfunction than surgery alone, shorter hormone deficiency-free survival and less frequent hormone recovery over the long term. The pathophysiology of radiation-induced hypopituitarism is complex and not very well understood. Several mechanisms have been proposed in the literature to elucidate the relation between irradiation and hypothalamic–pituitary dysfunction; this includes thalamic vascular damage with subsequent pituitary atrophy, microstructural change and axonal loss of the hypothalamus, and alterations of hypothalamic neurotransmitters with subsequent endocrine and metabolic disturbance [33–35]. In our centre, all patients are discussed in the pituitary multidisciplinary meeting to assess the need for surgery with careful consideration of the use of radiotherapy. We currently use proton beam therapy to treat children, teenagers and young adults up to their 25th birthday to reduce the late effects of radiation, according to the National Health Service (NHS) commissioning criteria. Recovery of pituitary dysfunction following treatment remains uncertain, with no apparent predictive clinical and radiological features. In this study, younger patients were more likely to regain normal endocrine function. Gender and postoperative evidence of residual tumour did not correlate with an overall recovery of pituitary hypofunction. This contrasts with the findings of Webb et al. [36], who reported better improvement in pituitary function in those with no tumour remanent. Of note, Little et al. [37] did not identify any predictors for regaining normal endocrine function. Berg et al. [38] assessed the recovery of GH and ACTH axes in 36 patients with pituitary disease after surgery using ITT and demonstrated a significant increase in GH and cortisol levels at 12 months following resection, with the recovery of both axes in 11% of those with GH deficiency and secondary adrenal insufficiency. Therefore, it is of crucial importance to provide a long-term endocrine follow-up for patients with pituitary disease following therapy to assess for a new hormone deficiency or recovery and to avoid unnecessary hormone replacement therapy. In our centre, we routinely perform full baseline and dynamic endocrine testing for GH and ACTH axes 6–8 weeks after surgery and on annual intervals when clinically indicated during follow-up after surgery and radiotherapy.

### Study strengths and weaknesses

This study included a large number of patients with NFPMs with a long follow-up duration making the finding generalisable. However, it is amenable to weaknesses of observational research, including data selection and loss. Although gonadotropin-expressing adenomas are commonly encountered in clinical practice, this study didn’t include other subtypes of silent adenomas like non-functioning somatotroph and lactotroph adenomas. Another limitation is that provocative testing was not performed in all patients; this may have caused some data bias. Levothyroxine therapy was only withdrawn when there was clinical and biochemical evidence of overreplacement; therefore, the incidence of TSH recovery might not have been fully assessed in this study.

## Conclusion

Non-functioning pituitary macroadenomas are associated with a significant degree of hypopituitarism at the time of diagnosis as well as after treatment. Both surgery and radiotherapy increase the risk of pituitary dysfunction. Hypopituitarism as a side effect of radiotherapy must be considered when this treatment is offered for tumour control.

Recovery of pituitary hormone deficit may occur post therapy. It is recommended to provide long term regular endocrine evaluation post-treatment to assess improvement in pituitary hypofunction and the need for long-term replacement therapy.
